# Genetic polymorphisms in early-onset myocardial infarction in a sample of Iraqi patients: a pilot study

**DOI:** 10.1186/s13104-020-05367-w

**Published:** 2020-11-24

**Authors:** Ameen M. Mohammad, Galawezh O. Othman, Chiman H. Saeed, Sarah Al Allawi, George S. Gedeon, Shatha M. Qadir, Nasir Al-Allawi

**Affiliations:** 1 Department of Internal Medicine, College of Medicine, University of Duhok, 9 Azadi Hospital Rd, Duhok, 1014AM Iraq; 2 Department of Biology, Education College, Salahaddin University, Erbil, Iraq; 3 Medical Research Center, Hawler Medical University, Erbil, Iraq; 4 Azadi Teaching Hospital, Duhok, Iraq; 5 Consultant Clinical Biochemist, Gedeon Medical Laboratories, Amman, Jordan; 6 Department of Hematology, Azadi Teaching Hospital, Duhok, Iraq; 7 Department of Pathology, College of Medicine, University of Duhok, Duhok, Iraq

**Keywords:** Premature myocardial infarction, MTHFR, ACE, PAI-1, HPA-1, β-fibrinogen, Risk factors, Iraq

## Abstract

**Objectives:**

Early-onset myocardial infarction constitutes nearly one third of cases of myocardial infarction among Iraqis, which is rather higher than the proportions reported in many Western countries. Thus this study was initiated to investigate the role of some genetic polymorphisms, as well as acquired risk factors in this condition.

**Results:**

A total of 102 Iraqi patients with first myocardial infarction aged 50 years, and 77 matched controls were enrolled. The DNAs of participants were screened for nine polymorphisms, namely: β-Fibrinogen (− 455G > A), Factor XIII (V34L), Plasminogen Activator inhibitor-1 (PAI-1, 4G/5G), Human Platelet Antigen-1 (HPA1a/b), 5,10-Methylenetetrahydrofolate Reductase MTHFR (C677T) and MTHFR (A1298C), Angiotensin-Converting Enzyme (ACE) 287 bp insertion/deletion (I/D), Apolipoprotein-B (ApoB: R3500Q), and Apolipoprotein-E (Apo E: E2/E3/E4), using PCR and reverse hybridization technique. Among traditional risk factors, univariate analysis revealed that smoking (OR 2.86 [95%CI 1.53–5.34]), hyperlipidemia (OR 5.23 [95%CI 2.66–10.29]), and diabetes mellitus (OR 4.05 [95% CI 1.57–10.41]) were significantly higher among patients compared to controls (P<0.001, <0.001 and 0.002 respectively), while none of the nine genetic polymorphisms reached significance. Multivariate Logistic regression, however, revealed that only smoking and hyperlipidemia retained significance (P of < 0.001 each). The need to initiate further studies on larger cohorts is paramount to understand the higher than expected frequency of early-onset myocardial infarction in our population.

## Introduction

Myocardial infarction (MI) is one of the leading causes of morbidity and mortality worldwide, whether in developed or developing countries [1]. Among Iraqis, early-onset myocardial infarction constitutes up to one third of all MI cases, well exceeding the 5–10% reported in developed countries [2–4]. The fact that there is documented familial clustering of MI cases suggests that in addition to the conventional acquired risk factors, there is an element of genetic predisposition, that may presumably be more relevant to younger patients [5, 6]. Throughout the past few decades a number of these genetic factors have been investigated by various researchers, but with conflicting results [5–9]. In the current study we assessed the roles of nine polymorphisms that may be linked to coronary artery disease. Among these polymorphisms are those at the Methylenetetrahydrofolate Reductase gene (MTHFR C677T and A1298C), which lead to a deficiency of this key enzyme with consequent hyperhomocysteinemia. The latter has been linked to arterial thrombosis [10]. Another polymorphism is factor XIII Val34Leu, which results in accelerated conversion of factor XIII to activated FXIIIa, and altered cross-linked fibrin clot structure [11]. While, β-Fibrinogen G -455 A involves the β-polypeptide chain of fibrinogen and leads to increased fibrinogen concentration, presumably conferring increased susceptibility to coronary heart disease [12]. Plasminogen activator inhibitor type 1 (PAI-1) is important in the regulation of endogenous fibrinolysis, and an increase in its circulating levels may be associated with the progression of atheromas and thrombosis [13]. A single nucleotide polymorphism of the GPIIIa gene causes Leu33Pro changing the human platelets antigen 1(HPA1) from 1a to 1b, with altered antigenic properties and an associated increase in platelet reactivity, thus fostering a prothrombotic state [14]. On the other hand, a polymorphism in the angiotensin-converting enzyme (ACE) gene characterized by insertion (I) or deletion (D) within its intron 16 has been described, with the D allele being associated with higher ACE levels and possible increased risk of coronary artery disease [15]. Apolipoprotein E (ApoE) is a glycoprotein that serves as a legend for cell surface receptor uptake of chylomicrons and Very Low Density Lipoprotein (VLDL) in the liver and extrahepatic cells [16]. ApoE polymorphism includes three isoforms E3, E2 and E4, among which the latter was more likely to be linked with coronary heart disease risk [17]. Moreover, Apolipoprotein B-100 (ApoB -100) is a component of the circulating low density lipoprotein (LDL) and serves as a legend for its uptake by the liver LDL receptors [18], and an arginine to glutamine change at codon 3500 of the ApoB-100 is a well- known genetic cause of hypercholesterolemia and premature atherosclerosis [19].

The current study is a pilot study focused on a population of young Iraqi adults with documented first incident of acute MI, to determine whether certain genetic polymorphisms are significant contributors to MI risk in this population.

## Main text

### Participants

A total of 179 participants who were 50 years or younger were enrolled. They included 102 patients with history of a single documented myocardial infarction visiting two cardiac centers in Erbil and Duhok-Iraq, together with 77 age and sex matched controls visiting the outpatient clinic at Azadi teaching hospital – Duhok-Iraq, but with no history of coronary artery disease or stroke.

### Methodology

All patients had their records reviewed, and a detailed history and clinical examination were undertaken in each. They also had the appropriate laboratory investigations. The patients' clinical examination and records were particularly scrutinized for the main traditional cardiovascular risk factors, namely smoking, hypertension, diabetes mellitus, and hyperlipidemia. Hypertension was defined as blood pressure in excess of the threshold of 140/90 mm Hg, and/or antihypertensive therapy use. Diabetes mellitus was defined as fasting serum glucose in excess of 126 mg/dL on two occasions, and/or anti-diabetic therapy use. Hyperlipidemia, and for the purposes of this study, was defined as a fasting serum cholesterol in excess of 200 mg/dL, and/or serum triglyceride in excess of 150 mg/dl, and/or the use of statins [20–22].

All patients and controls had their DNA extracted from EDTA blood by Qiagen QIAmp Kit (Qiagen, Germany). The extracted DNA was then amplified by multiplex Polymerase Chain Reaction (PCR) and reverse hybridized to detect the following mutations: β-Fibrinogen (-455 G > A), Factor XIII (V34L), Plasminogen Activator inhibitor-1 (PAI-1, Serpin E1, 4G/5G), Human Platelet Antigen-1 (HPA1a/b; GpIIIa; integrin beta 3 L33P), 5,10-Methylenetetrahydrofolate reductase MTHFR (C677T) and MTHFR (A1298C), Angiotensin-Converting Enzyme (ACE) 287 bp insertion/deletion (I/D), Apolipoprotein-B (ApoB: R3500Q), and Apolipoprotein-E (ApoE: E2/E3/E4), using the CVD StripAssay according to the manufacturer's instructions (ViennaLab Diagnostics GmbH, Austria).

### Statistical analysis

Data were evaluated using SPSS software (release 20; SPSS inc., Chicago, IL, USA). Univariate analysis utilized the student t-test for continuous variables, and the Chi square test for categorical ones, as appropriate. For associations with a P value < 0.25 by univariate analysis, multivariate analysis using logistic regression was used to evaluate the significance of the presence of at risk alleles and traditional risk factors, in patients compared to controls, with age and sex as covariates. A P value of < 0.05 was considered significant.

## Results and discussion

Among the 102 patients enrolled, the most frequent traditional risk factors encountered were hyperlipidemia and smoking, followed by hypertension, and diabetes mellitus (Table 1). Univariate analysis revealed that all these four risk factors had higher frequencies among patients compared to controls, and this was significant in all, except for hypertension. Multivariate analysis, on other hand, asserted the significance of both smoking and hyperlipidemia (P < 0.001 each). These observations are consistent with many previous studies worldwide on MI in young adults. In the INTERHEART study, which is a case control study, including around 15,000 patients and 15,000 controls from 52 countries, Yusuf and coworkers documented that smoking, hyperlipidemia, hypertension and diabetes had a greater relative effect on the risk of acute myocardial infarction in younger rather than older individuals [1]. Other studies reported that smoking is particularly more frequently associated with MI among young adults in various populations including Iranians, Americans, Omanis, Turks, Arabs in UAE and Indians [3, 23–27]. Similarly, hyperlipidemia has been documented as a highly important risk factor in young MI patients in several studies from various populations, including Indians, Arabs, Americans and West Europeans [1, 24, 26, 28].

**Table 1:** The frequencies of traditional risk factors in 179 enrolled patients and controls

Parameter	Patients no (%)	Controls no. (%)	P value	Odds ratio (95% confidence interval)
Number	102	77	–	–
Age mean (SD)	42.4 (6.19)	41.6 (7.09)	0.448	–
Sex (no. males/no. females)	77/25	57/20	0.823	–
Smoking	56 (54.9)	23 (29.8)	< 0.001	2.86 (1.53–5.34)
Hypertension	37 (36.3)	20 (26.0)	0.143	1.62 (0.85–3.11)
Diabetes mellitus	26 (25.5)	6 (7.8)	0.002	4.05 (1.57–10.41)
Hyperlipidemia	59 (57.8)	16 (20.8)	< 0.001	5.23 (2.66–10.29)

Among the nine polymorphism screened for by the current study, the highest allele frequencies were those for ACE (D allele), PAI (4G allele) and MTHFR 1298 (C allele) and MTHFR 677 (T allele). The ApoB R3500Q polymorphism was not detected in any of the patients or controls. The allele frequencies for majority of these polymorphisms were rather similar in patients and controls, except for β Fibrinogen -455 A allele which was more frequent among controls, and the HPA1 (1b allele) which was higher in patients (Table 2). Neither the latter two polymorphisms nor any of the other seven were significantly different between patients and controls, whether by univariate or multivariate analysis (Tables 2 and 3).

**Table 2:** The genotypes and allele frequencies of nine polymorphisms screened for among patients and controls (P values are for allele frequencies)

Mutation	Patients				Controls				P value
	Homo	Hetero	Wild	Allele frequency	Homo	Hetero	Wild	Allele frequency	
MTHFR C677T	13	33	56	28.9	12	25	40	31.8	0.554
MTHFR A1298C	20	48	34	43.1	16	29	32	39.6	0.503
FXIII Val34Leu	4	22	76	14.7	1	21	55	14.9	0.952
β Fibrinogen − 455 G > A	2	35	65	19.1	6	27	44	25.3	0.159
Plasminogen activator inhibitor (4G)	22	52	28	47.1	18	39	20	48.7	0.758
Human Platelet Antigen 1 (HPA1) 1b	4	27	71	17.1	1	14	62	10.4	0.07
Angiotensin-converting Enzyme (ACE D)	37	43	22	57.4	21	40	16	53.2	0.439
Apo B	0	0	0	0	0	0	0	0	–
Apo E allele (E4)	1	9	92	5.4	0	9	68	5.8	0.854

**Table 3:** Multivariate logistic regression in a model including variables with P < 0.25 by univariate analysis, with age and sex as covariates

Risk factor	B	SE	P value	Exp (B)	95% CI for Exp (B)
Fibrinogen GA	− 1.641	0.942	0.082	0.194	0.03–1.23
Fibrinogen AA	− 1.734	0.966	0.073	0.177	0.03–1.17
HPA-1 1a/1b	1.620	1.274	0.204	5.055	0.42–61.43
HPA-1 1b/1b	1.034	1.309	0.429	2.814	0.22–36.58
Smoking	1.438	0.404	< 0.001	4.214	1.91–9.30
Hypertension	0.021	0.397	0.959	1.021	0.47–2.22
Diabetes mellitus	0.934	0.554	0.092	2.544	0.86–7.53
Hyperlipidemia	1.469	0.391	< 0.001	4.347	2.02–9.36

Among the genetic polymorphisms investigated in the current study, two were in the MTHFR gene, namely: C677T and A1298G. Neither was found to be associated with increased risk of MI. This is contrast to an earlier study from the Iraqi population linking C677T to another type of arterial thrombosis, namely Ischemic stroke [29], and is also in contrast to studies from Turkey and Greece, suggesting that C677T mutation may be a risk factor for premature MI [30, 31]. However, our observations are consistent with other studies from Greece and South Africa which failed to document any associations [32, 33].

In the current study the allele frequency of FXIII Val34Leu polymorphism was not significantly different between patients and controls. This is consistent with some European studies on premature MI [34, 35], but is contrary to others, including a meta-analysis suggesting that it had an unexplained protective role in young MI patients as compared to healthy controls [36–38].

Studies on the association of β fibrinogen G-455A polymorphism with MI had revealed conflicting results. So that while a recent meta-analysis on Asian patients had linked it to a slightly increased risk [39], others failed to show such a link among Italian patients [40]. Moreover, and contrary to the latter two observations, a study on young Greek MI patients documented a protective effect of this polymorphism [41], which is consistent to some extent with our own observations, where both AA and AG genotypes conferred apparently protective effects by multivariate analysis, though this just failed to reach significance (Table 3). Such findings are unexpected, since this polymorphism is associated with increased fibrinogen, and thus a presumed increased risk of MI. One possible explanation may be that β fibrinogen G-455A polymorphism is in linkage disequilibrium with some other unknown polymorphism that confers a protective role. Further studies including larger numbers of patients are needed to address this issue.

A single insertion/deletion mutation in the promoter sequence of the PAI-1 gene at position -675 (4G/5G polymorphism) had been linked to PAI-1 levels with the 4G being associated with a higher enzyme level than the 5G allele [42, 43]. Nikolopoulos and coworkers in a meta-analysis demonstrated that the 4G allele is associated with a slightly increased risk of MI [44]. In the current study, and contrary to the latter meta-analysis, but similar to studies from Egyptian, young Italian and Finnish patients, the 4G allele was not associated with such an increased risk [7, 45, 46].

The results of studies on association of HPA 1b with premature MI are conflicting, so that while some studies dispute an association, others confirm it [47–49]. It is worth noting that the current study revealed that patients had a higher frequency of HPA 1b allele and were more likely to be homozygous to it than controls, but this did not reach significance (Tables 2 and 3).

Studies on Asian, Caucasian, as well as some North African populations documented that the ACE D allele is an MI risk factor [50–52]. The current study failed to document a significant association between ACE D allele and MI, which is consistent with studies from neighboring Iran, where it was concluded that ACE D allele is not an MI risk factor [53].

In relevance to APO E polymorphism, the current study did not show a significant difference between allele frequencies of E4 allele between patients and controls, which is consistent with a large meta-analysis including more than 22 000 British patients which also failed to document such a link [54]. While in relevance to APO B 100 polymorphism, the current study did not show any carriers of this mutation among 179 patients and controls enrolled, which is similar to studies from neighboring Turkey and Saudi Arabia where this mutation is also absent [55, 56]. This is in contrast to that seen among Caucasians, where this polymorphism is rather frequent and is an important contributor to MI risk [19].

In conclusion, it appears based on this pilot study that among young Iraqis with MI, traditional factors seem to be the main culprits related to risk, while none of the nine genetic polymorphisms studied were associated with increased risk.

## Limitations

The main limitation of the current study, as with all pilot studies is related to numbers of enrolled individuals, and more patients and controls would certainly have led to more informative results. However, certain important observations requiring scrutiny emerged, including a possible protective role for β fibrinogen G-455A polymorphism, and a possible increased risk of premature MI in association with HPA-1b polymorphism.

## Data Availability

All data sets used and/or analyzed during the current study are available from the corresponding author on reasonable request.

## Abbreviations

ACE: Angiotensin-Converting Enzyme.
ApoB: Apolipoprotein B.
ApoE: Apolipoprotein E.
HPA: Human Platelet Antigen.
LDL: Low-Density Lipoprotein
MI: Myocardial infarction.
MTHFR: Methylenetetrahydrofolate reductase.
PAI: Plasminogen Activator Inhibitor.
PCR: Polymerase Chain reaction.
VLDL: Very Low-Density lipoprotein
